# Granuloma, vasculitis, and demyelination in sarcoid neuropathy

**DOI:** 10.1111/ene.16091

**Published:** 2023-10-17

**Authors:** Naohiro Mouri, Haruki Koike, Yuki Fukami, Mie Takahashi, Satoru Yagi, Soma Furukawa, Masashi Suzuki, Yoshiyuki Kishimoto, Kenichiro Murate, Takamasa Nukui, Tamaki Yoshida, Yosuke Kudo, Mikiko Tada, Yuichi Higashiyama, Hirohisa Watanabe, Yuji Nakatsuji, Fumiaki Tanaka, Masahisa Katsuno

**Affiliations:** ^1^ Department of Neurology Nagoya University Graduate School of Medicine Nagoya Japan; ^2^ Department of Neurology Gifu Prefectural Tajimi Hospital Tajimi Japan; ^3^ Division of Neurology, Department of Internal Medicine Saga University Faculty of Medicine Saga Japan; ^4^ Department of Neurology Fujita Health University School of Medicine Toyoake Japan; ^5^ Department of Neurology, Faculty of Medicine University of Toyama Toyama Japan; ^6^ Department of Neurology Hiratsuka Kyosai Hospital Hiratsuka Japan; ^7^ Department of Neurology and Stroke Medicine Yokohama City University Graduate School of Medicine Yokohama Japan; ^8^ Department of Clinical Research Education Nagoya University Graduate School of Medicine Nagoya Japan

**Keywords:** sarcoid neuropathy, conduction block, demyelination, electrophysiology, granuloma, pathology

## Abstract

**Background:**

Despite the suggestion that direct compression by granuloma and ischemia resulting from vasculitis can cause nerve fiber damage, the mechanisms underlying sarcoid neuropathy have not yet been fully clarified.

**Methods:**

We examined the clinicopathological features of sarcoid neuropathy by focusing on electrophysiological and histopathological findings of sural nerve biopsy specimens. We included 18 patients with sarcoid neuropathy who had non‐caseating epithelioid cell granuloma in their sural nerve biopsy specimens.

**Results:**

Although electrophysiological findings suggestive of axonal neuropathy were observed, particularly in the lower limbs, all but three patients showed ≥1 abnormalities in nerve conduction velocity or distal motor latency. Additionally, a conduction block was observed in 11 of the 16 patients for whom waveforms were assessed; five of them fulfilled motor nerve conduction criteria strongly supportive of demyelination as defined in the European Academy of Neurology/Peripheral Nerve Society (EAN/PNS) guideline for chronic inflammatory demyelinating polyneuropathy (CIDP). In most patients, sural nerve biopsy specimens revealed a mild to moderate degree of myelinated fiber loss. Fibrinoid necrosis was observed in one patient, and electron microscopy analysis revealed demyelinated axons close to granulomas in six patients.

**Conclusions:**

Patients with sarcoid neuropathy may meet the EAN/PNS electrophysiological criteria for CIDP due to the frequent presence of conduction blocks. Based on our results, in addition to the ischemic damage resulting from granulomatous inflammation, demyelination may play an important role in the mechanism underlying sarcoid neuropathy.

## INTRODUCTION

Sarcoidosis is a systemic inflammatory disorder of unknown cause characterized by non‐caseating epithelioid cell granulomas. Sarcoidosis primarily affects the lungs, eyes, and skin, and the incidence of nervous system involvement is low (~5%) [1, 2, 3, 4, 5]. Cranial nerve palsy is the most common form of sarcoidosis with nervous system involvement (i.e., neurosarcoidosis), with bilateral facial nerve palsy being a typical manifestation [2]. Other lesions include parenchymal brain lesions, aseptic meningitis, spinal cord lesions, and skeletal muscle lesions [2, 3]. Peripheral nervous system involvement, excluding the involvement of cranial nerves, affects approximately 15% of patients with neurosarcoidosis [4, 5, 6]. Peripheral nervous system involvement is a factor associated with increased disability and mortality in patients with neurosarcoidosis [7], indicating the importance of the diagnosis and management of peripheral neuropathy in sarcoidosis. Research on sarcoid neuropathy involving the limbs has reported a wide range of clinical presentations, such as polyneuropathy, multiple mononeuropathy, radiculoneuropathy, and small fiber neuropathy [5, 8, 9, 10, 11, 12, 13]. These findings assertively demonstrate the diverse nature of this condition. Although previous studies on nerve biopsy specimens have suggested that direct compression of nerve fibers and vessels by granulomas and vasculitis causes nerve fiber damage [8, 11, 14], the pathomechanisms underlying sarcoid neuropathy have not been fully clarified.

Thus, to clarify this point, in the present study we examined the clinicopathological features of sarcoid neuropathy by focusing on electrophysiological and histopathological findings of sural nerve biopsy specimens.

## PATIENTS AND METHODS

### Patients

We retrospectively investigated 18 consecutive patients with sarcoid neuropathy who were referred to Nagoya University Graduate School of Medicine from 2000 to 2022 and had a non‐caseating epithelioid cell granuloma in sural nerve biopsy specimens (Table 1). There were 5 men and 13 women, 52–84 years of age (69.4 ± 8.0 years, mean ± SD). The diagnosis of sarcoidosis was made according to the criteria proposed by the Japanese Ministry of Health, Labor, and Welfare of Japan and the Japanese Society of Sarcoidosis and Other Granulomatous Disorders in 2015 [15]. We reviewed clinical and neurological assessments, routine blood and cerebrospinal fluid analyses, and nerve conduction studies. Patients were excluded when an alternative diagnosis was considered more likely or when having other diseases that might influence pathological findings, such as diabetes mellitus. Patients did not receive immunomodulatory therapies at the time of nerve biopsy, except for Patient 2 who was diagnosed with sarcoidosis due to spinal cord involvement.

**TABLE 1:** Clinical characteristics of patients.

Case	Sex	Age at biopsy (years)	Initially affected organ	Duration from neuropathy onset to biopsy	Initial symptom of neuropathy	Pattern of neuropathy	Motor involvement	CSF findings	
								Cell (*n*/mm^3^)	Protein (mg/dL)
1	F	52	Peripheral nerve	2 months	N	MM	−	ND	ND
2	F	74	Spinal cord	ND^a^	ND^a^	MM	−	11	73
3	F	76	Lung	13 months	P	MM	−	10	56
4	F	70	Peripheral nerve	6 months	N	PN	−	13	60
5	F	66	Peripheral nerve	7 years	N	PN	+	1	27
6	F	56	Eye	9 months	N, W	MM	+	ND	ND
7	F	62	Lung	2 months	N	MM	+	7	48
8	M	68	Peripheral nerve	11 months	N	MM	+	ND	ND
9	F	76	Eye	5 years	N	PN	+	ND	ND
10	F	84	Peripheral nerve	11 months	N	MM	−	2	38
11	F	73	Peripheral nerve	8 months	N	MM	+	2	39
12	M	67	Peripheral nerve	10 months	N	MM	+	12	63
13	M	69	Eye	10 months	N	MM	+	1	61
14	F	78	Eye	3 months	N	PN	+	3	25
15	M	78	Eye	9 months	P	MM	+	2	53
16	F	65	Peripheral nerve	1 month	N	MM	+	6	44
17	M	64	Peripheral nerve	1 months	N, W	PN	+	6	62
18	F	71	Peripheral nerve	7 months	N	MM	+	2	46

Abbreviations: −, absent; +, present; MM, multiple mononeuropathy; N, numbness; ND, not determined; P, pain; PN, polyneuropathy; W, weakness.

^a^ Not determined due to the presence of myelopathy.

This study conformed to the Ethical Guidelines for Medical and Biological Research Involving Human Subjects endorsed by the Japanese Government and was approved by the Ethics Review Committees of Nagoya University Graduate School of Medicine. All patients provided their written informed consent.

### Electrophysiological assessments

Motor and sensory conduction were measured using standard methods with surface electrodes for stimulation and recording just before sural nerve biopsy (14.5 ± 22.4 months from the onset of neuropathy) [16]. Motor conduction was tested in the median, ulnar, tibial, and peroneal nerves by recording from the abductor pollicis brevis, abductor digiti minimi, abductor hallucis brevis, and extensor digitorum brevis, respectively. The following nerve segments were used to calculate the motor nerve conduction velocity (MCV): from the wrist to the elbow for the median nerve, from the wrist to the distal portion of the elbow for the ulnar nerve, from the ankle to the popliteal fossa for the tibial nerve, and from the ankle to the distal portion of the fibular head for the peroneal nerve. Sensory conduction was investigated in the median, ulnar, and sural nerves using an antidromic recording from ring electrodes located at the second and fifth digit for the median and ulnar nerves, respectively, and bar electrodes at the ankle for the sural nerve. In addition, the sensory nerve conduction velocity (SCV) was calculated for the distal segment. Amplitudes of compound muscle action potentials (CMAP) and sensory nerve action potentials (SNAP) were measured from the baseline to the first negative peak. We investigated the presence of electrophysiological findings supportive of demyelination based on the nerve conduction criteria of CIDP defined in the European Academy of Neurology/Peripheral Nerve Society (EAN/PNS) guideline [17]. Given that Japanese individuals are vulnerable to peroneal nerve injury owing to their unique lifestyle habits, such as sitting on the floor [18], only conduction block and temporal dispersion were assessed for the peroneal nerve.

Control values were obtained in the same manner from 63 healthy volunteers who were evaluated in the electrophysiology laboratory of Nagoya University Graduate School of Medicine (67.4 ± 6.3 years of age, mean ± SD; 30 men, 33 women) [19]. Values that deviated by ±2 SD from the mean of the normal control values were considered abnormal (i.e., upper/lower limit of normal values). These values were <50.6, <46.7, and <38.1 m/s for the MCV; >4.3, >3.2, and >5.4 ms for the distal motor latency (DML) of the median, ulnar, and tibial nerves; and <44.9, <42.7, and <39.0 m/s for the SCV of the median, ulnar, and sural nerves, respectively.

### Pathological assessment of sural nerve biopsy specimens

Sural nerve biopsies were performed as previously described [20]. Then, the specimens were divided into two groups. The first group of specimens was fixed in 2.5% glutaraldehyde in 0.125 M cacodylate buffer (pH 7.4) and embedded in epoxy resin for morphometric and electron microscopic studies. The density of myelinated fibers was assessed in toluidine blue‐stained semi‐thin sections using a computer‐assisted image analyzer (Luzex FS; Nikon), and the density of small and large myelinated fibers was calculated as previously described [20]. For electron microscopy, epoxy resin‐embedded specimens were cut into 70 nm‐thick ultrathin sections and stained with uranyl acetate and lead citrate. Then, a fraction of the glutaraldehyde‐fixed sample was processed for a teased‐fiber study, and pathological conditions were microscopically assessed according to previously described criteria [21]. Control values were obtained from seven autopsy cases (67.7 ± 8.9 years of age, mean ± SD; 3 men, 4 women) who died of non‐neurological diseases [19].

The second group of specimens was fixed in a 10% formalin solution and embedded in paraffin. Sections were cut by routine methods and stained with hematoxylin and eosin and the Masson trichrome method. To confirm the granuloma distribution, immunohistochemistry was performed on deparaffinized sections via immunoperoxidase staining using a mouse anti‐human CD68 monoclonal antibody (Dako).

### Statistical analysis

All analyses were performed with StatView version 5.0 (SAS Institute). Quantitative data are presented as the mean ± SD. Statistical analyses were performed using the Fisher exact test or the Mann–Whitney *U* test as appropriate. Values of *p* < 0.05 were considered significant.

## RESULTS

### Clinical characteristics

The first organ involved was the peripheral nerve in 10 patients, the eye in five, the lung in two, and the spinal cord in one. The duration from neuropathy onset to sural nerve biopsy ranged from 2 months to 7 years. All but two patients initially complained of numbness in the limbs. Patients 3 and 15 reported pain as their initial symptom of neuropathy. The sensory deficits in 13 patients corresponded to mononeuritis multiplex, while five patients had polyneuropathy (Patients 4, 5, 9, 14, and 17). Secondarily, four patients (Patients 3, 9, 15, and 17) complained of pain in the affected region and 13 exhibited apparent weakness in the limbs at the time of sural nerve biopsy (Table 1).

### Electrophysiological features

There was a significant reduction of CMAP and SNAP amplitude in the lower limbs (*p* < 0.0001 for the tibial nerve CMAP and the sural nerve SNAP) (Table 2).

**TABLE 2:** Results from nerve conduction studies.

Case	Median nerve		Ulnar nerve		Tibial nerve	Sural nerve
	Motor	Sensory	Motor	Sensory	Motor	Sensory
	MCV/DL/CMAP	SCV/SNAP	MCV/DL/CMAP	SCV/SNAP	MCV/DL/CMAP	SCV/SNAP
	(m/s)/(ms)/(mV)	(m/s)/(μV)	(m/s)/(ms)/(mV)	(m/s)/(μV)	(m/s)/(ms)/(mV)	(m/s)/(μV)
1	51.8/3.1/7.7	46.4/8.0	44.7/2.4/7.5	44.8/16	37.2/5.5/3.17	NE
2	60.7/4.1/3.7	60.7/25.5	50.6/2.7/6.2	53.2/19.8	42.7/4.3/10.8	46.6/3.6
3	49/4.7/4.28	44/13	47/3.1/8.51	46/23	31/9.2/1.18	39/3.0
4	64.3/4.14/10.5	58.3/11.0	67.6/3.02/7.81	63.7/6.9	38.1/4.7/4.51	48.2/3.51
5	51.9/3.45/6.4	45.1/8.9	53.8/2.49/5.1	50.0/7.9	47.2/5.61/6.39	44.7/10.0
6	53.9/3.28/5.26	54.6/14	48.2/2.74/3.98	58.4/14.3	44.4/3.03/1.8	56.5/3.7
7	46/ND/6.7	53/ND	ND	ND	37.8/ND/4.4	43/0.8
8	48.7/3.5/7.6	63.4/10.9	49.8/3.10/5.6	52.4/8.5	33.5/4.2/5.1	46.3/1.5
9	54/3.8/5.6	48/11.0	58/2.8/6.0	59/15.9	42.0/4.8/5.7	48.0/1.3
10	57/4.4/6.3	50/10.5	63/2.5/10.4	59/12.0	25/5.5/1.6	28/4.0
11	57/3.2/7.9	54.3/25.6	63.7/2.6/7.4	59.5/20.3	34.3/4.2/1.5	51.5/2.8
12	34.9/4.41/4.92	43.5/1.7	54.7/3.09/4.64	44.4/1.7	54.7/3.09/4.64	44.4/2.7
13	60.6/3.78/11.3	45.3/12.8	58.0/2.43/12.3	50.0/20.7	33.9/4.05/11.7	42.0/3.4
14	46.6/3.70/6.2	50.8/29.7	40.6/3.5/7.4	51.8/29.27	40.6/3.7/7.40	47.0/7.7
15	52.8/4.44/7.59	53.8/21.6	55.6/3.48/7.38	52.1/18.5	39.3/5.01/4.16	40.6/3.10
16	38.4/3.98/4.98	35.7/0.80	30.2/2.91/4.89	NE	NE	NE
17	43.3/4.00/11.5	43.0/3.0	39.1/3.50/6.9	NE	31.9/5.10/2.41	NE
18	42.8/4.20/6.5	42.6/1.3	45.6/3.2/6.4	45.3/19.1	31.3/4.8/1.5	NE
Mean	50.8***/3.9**/6.9	49.6**/12.3****	51.2*/2.9**/7.0	52.6/14.0	38.9***/4.8*/4.6****	46.1*/2.7****
Controls	56.8/3.5/ 7.5	55.3/22.3	57.1/2.6/6.9	52.7/18.7	44.7/4.0/10.8	49.2/12.8

*Notes*: Nerve conduction studies were performed just before sural nerve biopsy. Control values were obtained from 63 normal volunteers (67.4 ± 6.3 years of age, mean ± SD; 30 men, 33 women) [19].

Abbreviations: CMAP, compound muscle action potentials; DL, distal latency; MCV, motor nerve conduction velocity; ND, not determined; NE, not elicited; SCV, sensory nerve conduction velocity; SNAP, sensory nerve action potentials.

* *p* < 0.05.

** *p* < 0.01.

*** *p* < 0.001.

**** *p* < 0.0001 (Mann–Whitney *U* test).

**FIGURE 1:**
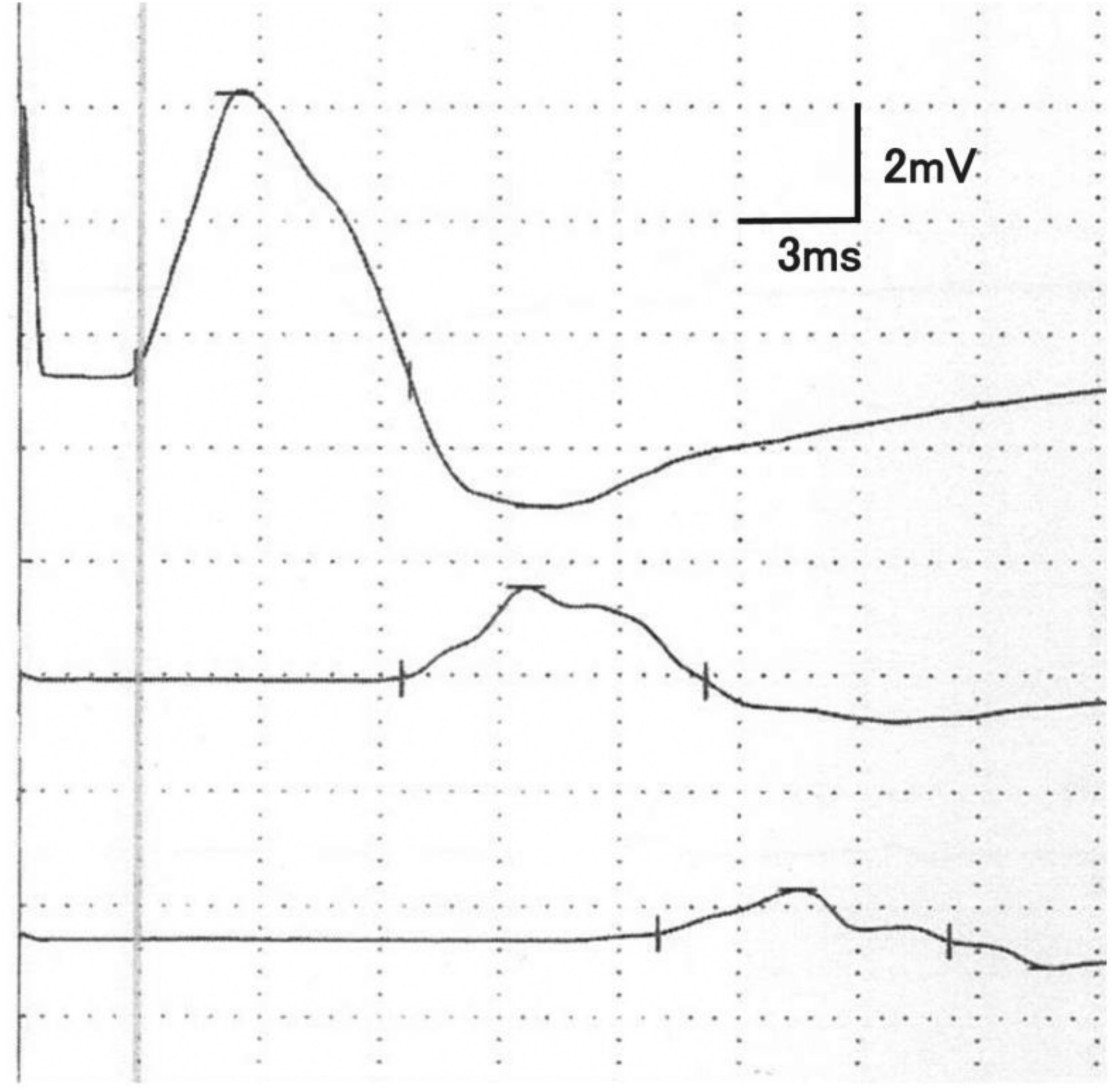
Representative waveform in the ulnar nerve showing a conduction block. The amplitude of the compound muscle action potential at the proximal part was markedly decreased compared with that at the distal part.

Additionally, many patients exhibited findings suggestive of demyelination in the upper and lower limbs. Patients 10, 12, and 16 showed a ≥30% reduction below the MCV lower limit of normal for the tibial, median, and ulnar nerves, respectively. In addition, Patient 3 exhibited a ≥50% prolongation above the DML upper limit of normal for the tibial nerve. Of the 16 patients whose waveforms were assessed, 11 (69%) patients (Patients 1, 3–5, 9, 12–14, and 16–18) showed a conduction block in the median (Patients 12 and 18), ulnar (Patients 1, 3, 5, 14, and 16–18), or peroneal (Patients 4, 9, 12–14, and 18) nerve (Figure 1 and Table S1). Temporal dispersion was observed in four patients (Patients 3, 12, 14, and 18). Notably, given the presence of conduction block in two nerves, four of these patients (Patients 5, 12, 14 and 18) fulfilled the motor nerve conduction criteria strongly supportive of demyelination, as defined in the EAN/PNS guideline for CIDP [17]. Another patient (Patient 3) also fulfilled these criteria owing to the presence of conduction block in one nerve and ≥1 other demyelinating parameters in ≥1 other nerve.

Although motor nerve conduction criteria of CIDP were not satisfied, a trend toward signs of demyelination was observed: MCV was in the abnormal range in 8 of 18 (44%), 5 of 17 (29%), and 10 of 17 (59%) patients, whereas distal latency was abnormal in 3 of 17 (18%), 3 of 17 (18%), and 3 of 16 (19%) patients for the median, ulnar, and tibial nerves, respectively. SCV was in the abnormal range in 5 of 18 (28%), 0 of 14 (0%), and 1 of 14 (7%) patients, respectively for the median, ulnar, and sural nerves. All patients, except for three (Patients 2, 6, and 9), showed ≥1 abnormalities in these nerve conduction indices. The concomitant abnormalities in distal latency and SCV values in the median nerve observed in Patients 3 and 12 might be due to carpal tunnel syndrome. However, these patients did not report typical symptoms and signs resulting from increased pressure at the wrist, such as pain at night, weakness of hand muscles, and positive tunnel signs.

### Pathological findings in the sural nerve

**FIGURE 2:**
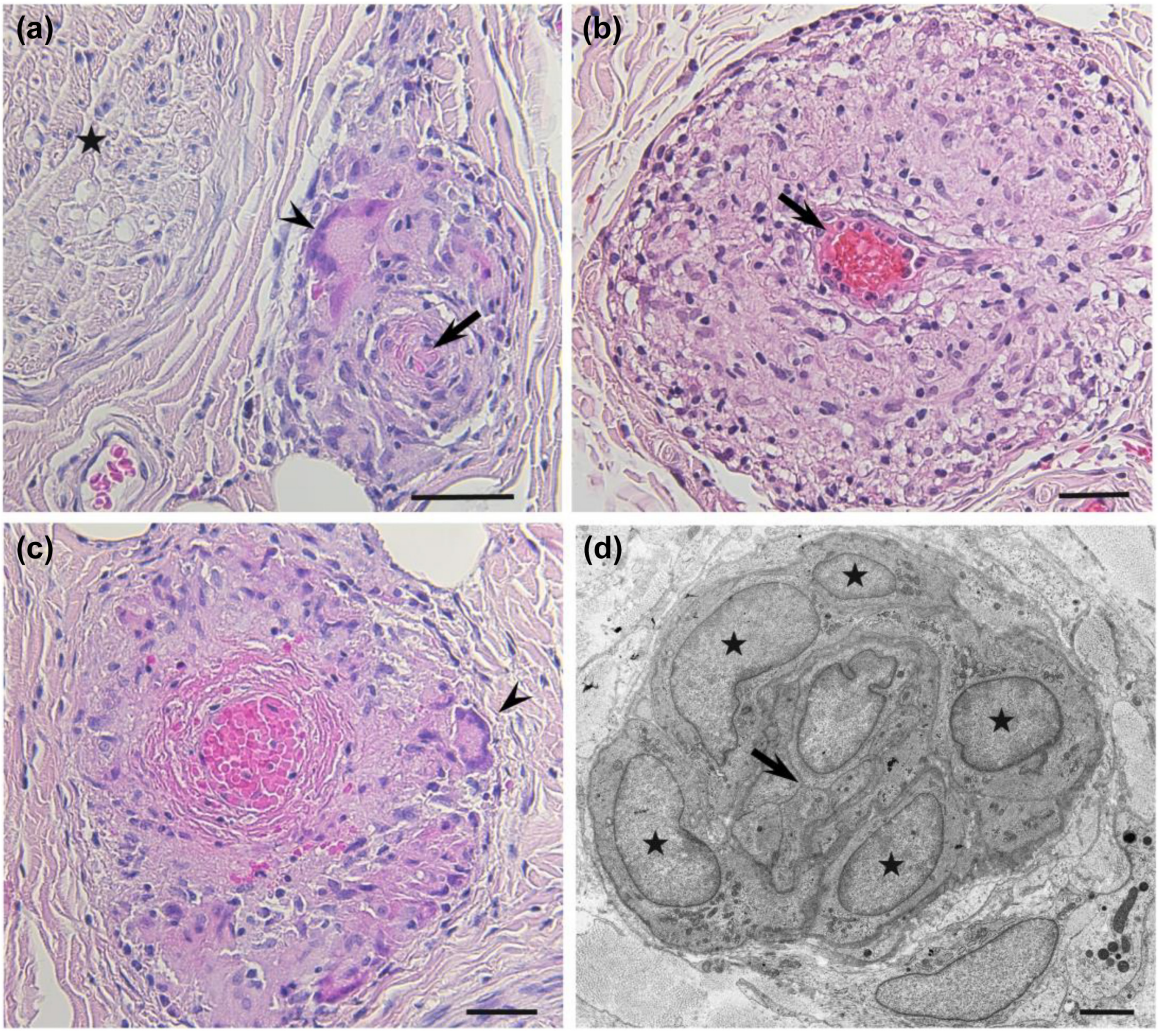
Representative pathological findings in sural nerve biopsy specimens from patients with sarcoid neuropathy. (a) Distribution of granulomas in the epineurium in association with vessel location. An epineurial vessel indicated by an arrow is surrounded by a granuloma with a multinucleated giant cell (arrowhead). The vascular lumen is occluded. An asterisk indicates the endoneurium. (b) Extensive granuloma formation around a vessel in the epineurium. An arrow indicates the vascular lumen. (c) Fibrinoid necrosis of the epineurial vessel in a patient with extensive granuloma formation. An arrowhead indicates a multinucleated giant cell. (d) On electron microscopy, some epineurial microvessels were surrounded by macrophages (asterisks). The vascular lumen is occluded (arrow). Cross sections. Hematoxylin and eosin staining (a–c). A sample prepared for transmission electron microscopy (d). Scale bars = 30 μm (a–c) and 2 μm (c).

Non‐caseating epithelioid cell granulomas with or without multinucleated giant cells were observed in the epineurium in all patients (Table 3). The distribution of epithelioid cell granulomas in the epineurium tended to be associated with vessel location (Figure 2a,b). In fact, the walls of severely affected vessels were replaced by granulomas. In addition, narrowing or occlusion of the vascular lumen was observed in these vessels. There was obvious epineurial vessel occlusion in six patients. One of these patients (Patient 8), who showed extensive granuloma formation, had fibrinoid necrosis of the epineurial vessel in association with granulomatous lesions (Figure 2c). Clusters of mononuclear cells, including CD‐68‐positive macrophages, but without obvious granulomatous lesions were occasionally observed around small vessels. On electron microscopy, some epineurial microvessels were surrounded by macrophages (Figure 2d), supporting that the granulomas in the epineurium are formed along with vessels.

**TABLE 3:** Pathological findings in sural nerve biopsy specimens.

Case	Myelinated fiber density (*n*/mm^2^)	Segmental de/re‐myelination (%)	Axonal degeneratio*n* (%)	Focal nerve fiber loss	Distribution of epithelioid granuloma			Morphology of epineurial vessels	
					Epineurium	Perineurium	Endoneurium	Fibrinoid necrosis	Occlusion
1	6148	2.0	0.6	−	+	+	+	−	+
2	7196	12.4	23.7	+	+	+	−	−	−
3	5340	2.7	19.3	−	+	−	+	−	−
4	3481	2.7	40.3	+	+	−	−	−	−
5	8035	4.2	0.6	−	+	−	−	−	+
6	6512	1.2	16.9	−	+	+	+	−	−
7	6754	0.9	0.5	−	+	+	+	−	+
8	6858	ND	ND	+	+	+	+	+	+
9	7170	0.4	3.2	−	+	+	−	−	−
10	3889	10.6	14.5	−	+	+	−	−	−
11	6035	2.9	13.7	+	+	−	−	−	−
12	5975	7.2	40.2	+	+	−	−	−	−
13	5871	9.7	23.4	−	+	−	−	−	−
14	5474	19.0	4.9	−	+	−	−	−	−
15	6322	9.7	23.4	+	+	+	−	−	−
16	4374	15.1	10.5	+	+	+	+	−	+
17	1412	13.2	5.7	−	+	−	−	−	+
18	3420	15.5	2.8	−	+	+	+	−	−
Mean ± SD	5570 ± 1666	7.6 ± 6.0	14.4 ± 12.8						
Controls^a^	7492 ± 1168	11 ± 6	2 ± 2						

Abbreviations: −, absent; +, present; ND, not determined.

^a^ Control values were obtained from seven autopsy cases in which the patients died of non‐neurological diseases (67.7 ± 8.9 years of age, mean ± SD; 3 men, 4 women) [19].

**FIGURE 3:**
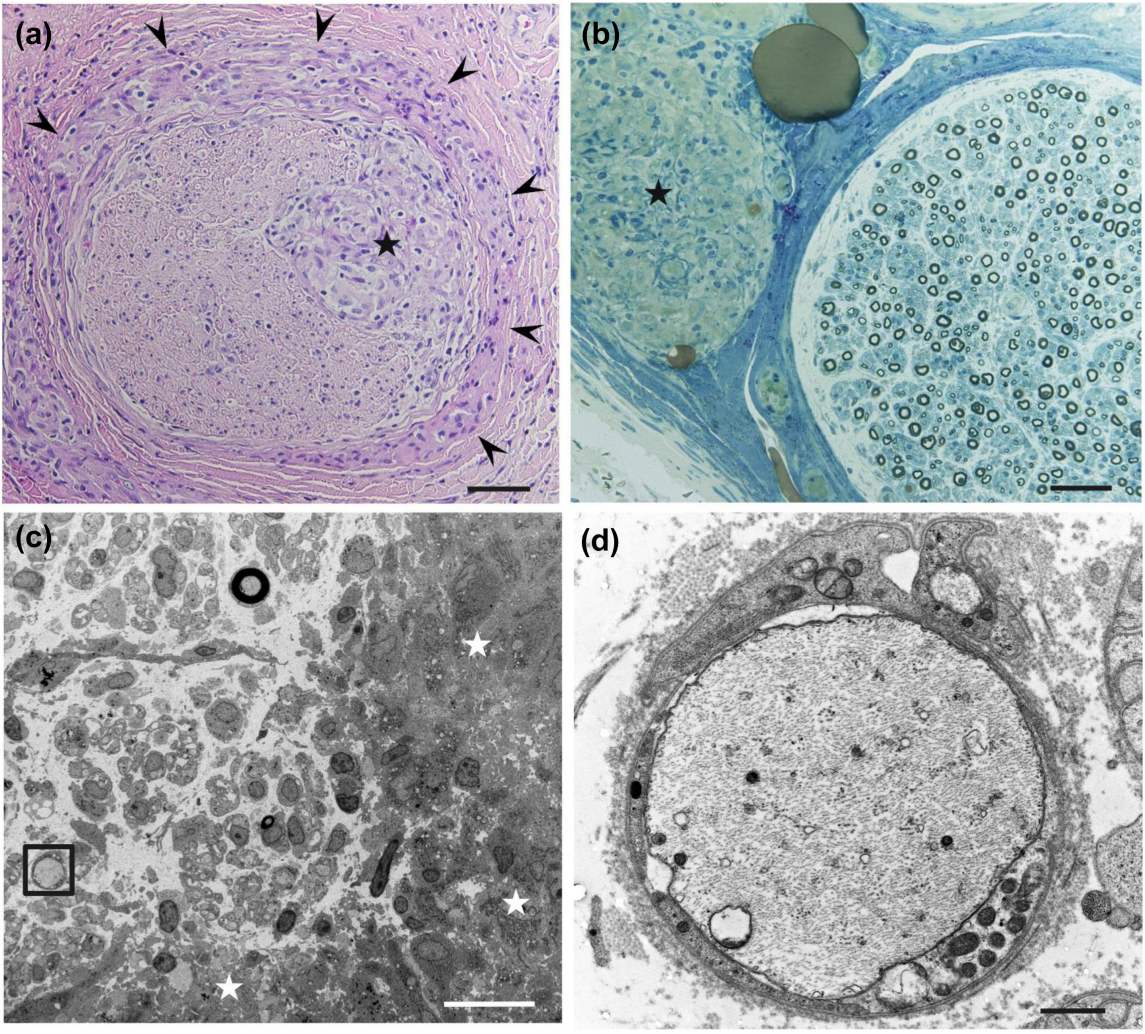
Granulomas and myelinated fibers in the endoneurium. (a) Many granulomas in the endoneurium (asterisk) were continuous with or adjacent to perineurial granulomas, as indicated by arrowheads. (b) Myelinated fibers relatively preserved with respect to the extent of granuloma formation. A granuloma in the epineurium is indicated by an asterisk. (c) On electron microscopy, a disproportionately large unmyelinated axon (indicated by a box), considered to have been originally myelinated, in the vicinity of the granulomas indicated by asterisks. (d) High‐powered view of the region within the box in (c). Cross sections of sural nerve biopsy specimens from patients with sarcoid neuropathy. Hematoxylin and eosin staining (a) and toluidine blue staining (b). A sample prepared for transmission electron microscopy (c, d). Scale bars = 50 μm (a, b), 20 μm (c), and 1 μm (d).

In addition, granulomas were observed at the perineurium in 10 (56%) patients and at the endoneurium in 7 (39%) patients. Many granulomas in the endoneurium were either continuous with or adjacent to perineurial granulomas (Figure 3a); the granulomas appeared to invade the endoneurium from the perineurium, particularly via the intrafascicular septum continuous with the perineurium.

The extent of myelinated fiber loss was mild to moderate in most patients despite the conspicuous granulomatous lesions (Figure 3b). There was apparent focality of nerve fiber loss in seven patients (Patients 2, 4, 8, 11, 12, 15, and 16). The total density of myelinated fibers was 5570 ± 1666 fibers/mm^2^, 74% of the mean control value (*p* < 0.01). Axonal sprouting of myelinated fibers suggestive of axonal regeneration was observed at the sites of myelinated fiber loss in Patients 11 and 16. In teased‐fiber preparations, the frequency of axonal degeneration was significantly higher than in normal controls (14.4% ± 12.8% vs. 2.4% ± 1.7%, *p* < 0.05), while the proportion of segmental de/re‐myelination was similar to normal controls (7.6% ± 6.0% vs. 10.7% ± 5.6%).

On electron microscopy, disproportionately large unmyelinated axons, >3 μm in diameter, were found in the vicinity of granulomas in six patients (Patients 1, 3, 6, 8, 16, and 18; Figure 3c,d). These axons might have been myelinated at first but had undergone demyelination [22]. One of these patients (Patient 18) showed a demyelinated axon within the granuloma. Patient 6 showed a myelinated fiber, with the cytoplasm located between layers of myelin lamellae (Figure S1). This cytoplasm was similar to that of a macrophage, given that it contained folded cytoplasmic processes and myelin debris [23, 24]. Although we did not observe the typical onion bulb structures composed of multiple layers of flattened Schwann cell processes, two patients (Patients 16 and 18) had myelinated fibers incompletely surrounded by one layer of flattened Schwann cell processes.

## DISCUSSION

The clinical presentation of neuropathy associated with sarcoidosis is diverse, including cranial neuropathy, polyneuropathy, multiple mononeuropathy, radiculoneuropathy, small fiber neuropathy, and autonomic neuropathy [5, 8, 9, 10, 11, 12, 13]. This diversity of neuropathic presentations may indicate a complicated pathophysiology, involving both granulomatous and non‐granulomatous inflammatory processes [25]. Although the mechanisms of nerve fiber damage resulting from granulomatous inflammation have not been fully elucidated, previous studies of nerve biopsy specimens suggested direct compression of nerve fibers by granulomas and ischemia resulting from the compression by granulomas or necrotizing vasculitis [8, 11, 14].

In this study, we investigated the clinicopathological characteristics of neuropathy in patients with sarcoidosis by focusing on electrophysiological and histopathological findings of sural nerve biopsy specimens. In the epineurium of the sural nerve, the location of granulomatous inflammation was mostly associated with vascular walls. Although necrotizing vasculitis has been reported in patients with sarcoid neuropathy [8, 11], fibrinoid necrosis was found in only one patient in our study. This scarcity of fibrinoid necrosis is in contrast with other diseases characterized by necrotizing vasculitis, particularly anti‐neutrophil cytoplasmic antibody (ANCA)‐associated vasculitis [26, 27]. However, narrowing and occlusion of the vascular lumen from mechanical compression due to extensive granuloma formation were often observed in our patients. These findings may indicate that the ischemia caused by vasculopathy due to granulomatous inflammation is milder than that caused by other ordinary necrotizing vasculitic neuropathies. In fact, the neuropathic features in our patients were somewhat different from those of other ordinary vasculitic neuropathies, including ANCA‐associated vasculitis and non‐systemic vasculitic neuropathy, in terms of its longer disease duration and less frequent focal involvement (i.e., mononeuritis multiplex) and pain [28]. With respect to pathology, the focal nerve fiber loss observed in seven (39%) of our patients is compatible with ischemic damage resulting from vasculitis. However, the extent of nerve fiber loss in our patients was also relatively milder than that in patients with ANCA‐associated vasculitis or non‐systemic vasculitic neuropathy [26, 28, 29].

Another characteristic histopathological finding in our study was demyelinated axons, found in five (28%) patients and located close to or within granulomas. Previous studies reported aberrant nerve conduction parameters suggestive of demyelination in patients with sarcoid neuropathy [8, 10, 30, 31]. Many of our patients also showed slower MCV and SCV and DML prolongation. Additionally, a conduction block was observed in 11 of the 16 (69%) patients whose waveforms were assessed. Such a conduction block may suggest the presence of focal lesions in the middle portion of the nerve trunk. These results align with those of previous studies [30, 31]. In addition, a conduction block has been reported in patients with vasculitic neuropathy [32, 33]. However, in such cases, it appears only in the early stages of axonal degeneration resulting from ischemia, and serial nerve conduction studies show the decrease in CMAP amplitude not only in the proximal but also in the distal portion, reflecting severe axonal loss [33]. In contrast, the conduction block in sarcoid neuropathy resolves by an increased CMAP amplitude at the distal portion after corticosteroid therapy, suggesting that focal lesions are reversible [30, 31]. In the present study, five nerves (three ulnar and two peroneal nerves) from four patients (Patients 13, 14, 16, and 17) had follow‐up data. Of these, one peroneal nerve (Patient 13) and one ulnar nerve (Patient 14) showed an improvement in the conduction block after the initiation of immunomodulatory therapies. Therefore, in addition to reversible ischemia resulting from compression caused by granulomas around vessels and perineurium, demyelination might also be involved in the conduction block in patients with sarcoid neuropathy.

The mechanisms inducing demyelination in sarcoid neuropathy have not yet been clarified. Segmental demyelination in teased‐fiber preparations, similar to that reported in patients with Guillain–Barré syndrome or CIDP [34, 35], was demonstrated in a patient with sarcoid neuropathy accompanied by reversible conduction block [31]. In Guillain–Barré syndrome and CIDP, demyelination results from selective phagocytosis of myelin by macrophages [23, 24]; although we found one myelinated fiber showing similar findings in Patient 6, such a lesion was not found in other patients. The presence of demyelination, despite the absence of phagocytosis of myelin by macrophages, in sarcoidosis was similar to that in neurolymphomatosis [36]. As the location of demyelination was associated with granulomas in our study, the production of humoral factors, such as matrix metalloproteinases, induced by granulomatous inflammation may cause myelin degradation. Alternatively, mechanical stress, such as compression or distortion of myelinated fibers by granulomas, may induce demyelination. Further research is needed to clarify the mechanisms underlying sarcoid neuropathy from the viewpoint of granulomatous lesions.

## AUTHOR CONTRIBUTIONS

**Naohiro Mouri:** Investigation; Writing – original draft. **Haruki Koike:** Conceptualization; Investigation; Funding acquisition; Writing – original draft; Methodology; Validation; Visualization; Software; Formal analysis; Data curation. **Yuki Fukami:** Investigation; Writing ‐ review & editing. **Mie Takahashi:** Investigation; Writing – review & editing. **Satoru Yagi:** Investigation; Writing – review & editing. **Soma Furukawa:** Investigation; Writing – review & editing. **Masashi Suzuki:** Investigation; Writing – review & editing. **Yoshiyuki Kishimoto:** Investigation; Writing – review & editing. **Kenichiro Murate:** Investigation; Writing – review & editing. **Takamasa Nukui:** Investigation; Writing – review & editing. **Tamaki Yoshida:** Investigation; Writing – review & editing. **Yosuke Kudo:** Investigation; Writing – review & editing. **Mikiko Tada:** Investigation; Writing – review & editing. **Yuichi Higashiyama:** Investigation; Writing ‐ review & editing. **Hirohisa Watanabe:** Investigation; Writing ‐ review & editing. **Yuji Nakatsuji:** Investigation; Writing ‐ review & editing. **Fumiaki Tanaka:** Investigation; Writing ‐ review & editing. **Masahisa Katsuno:** Investigation; Writing ‐ review & editing; Supervision.

## FUNDING INFORMATION

This work was supported in part by the Health and Labour Sciences Research Grant on Intractable Diseases (Neuroimmunological Diseases) from the Ministry of Health, Labour and Welfare of Japan (20FC1030) and JSPS KAKENHI Grant Number 20K07882.

## CONFLICT OF INTEREST STATEMENT

None declared.

## Supporting information


Figure S1:

Table S1.

## Data Availability

The data that support the findings of this study are available on request from the corresponding author. The data are not publicly available due to privacy or ethical restrictions.
