# Supplementary figures and images for: Granuloma, vasculitis, and demyelination in sarcoid neuropathy
Source: Eur J Neurol. 2023 Oct 17;31(1):e16091. doi: 10.1111/ene.16091 (PMC11235865; doi:10.1111/ene.16091)

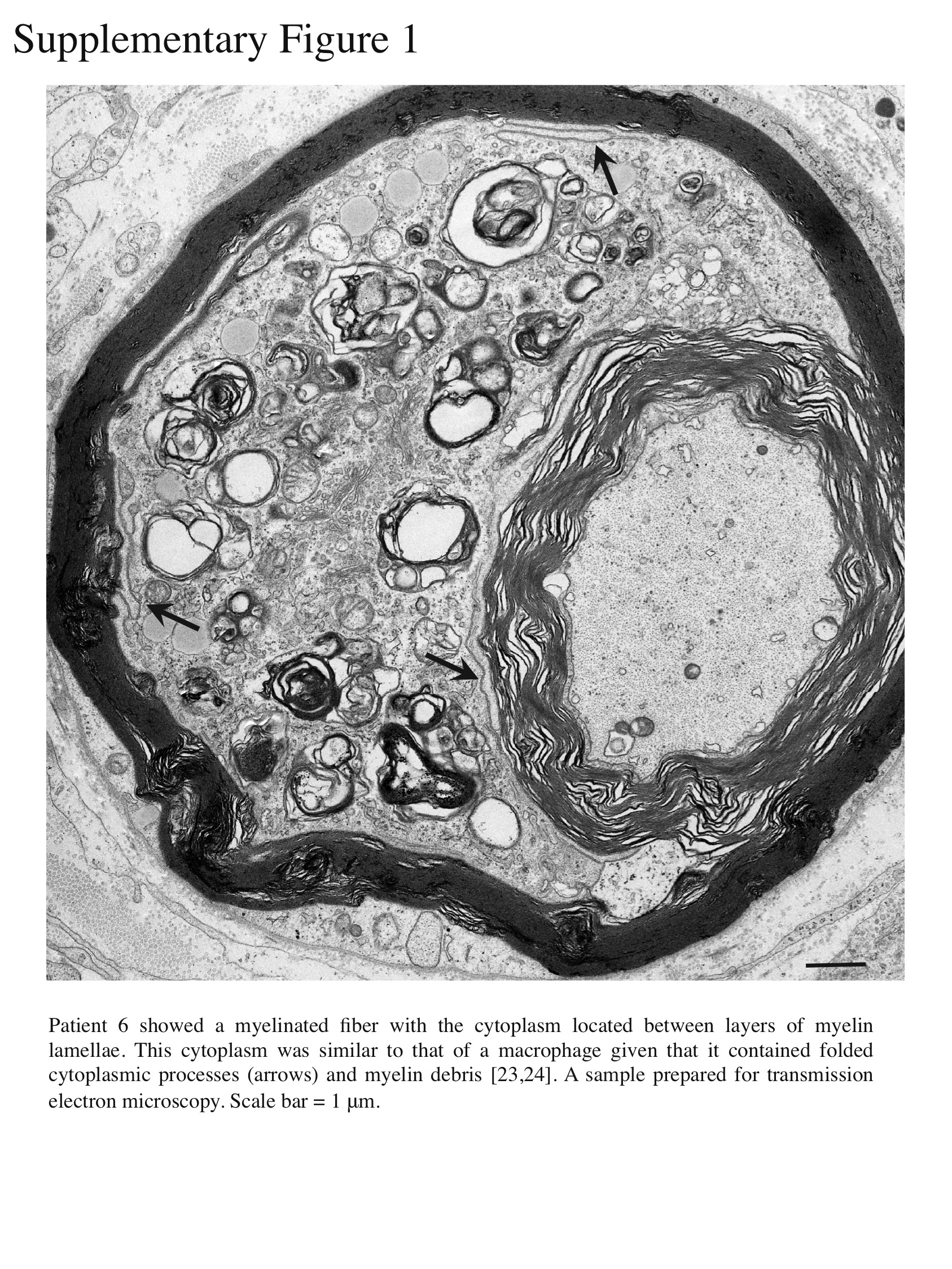

Supplement: Supplementary file 1 — Figure S1: [file ENE-31-e16091-s001.jpg]
