# Supplementary material for: Granuloma, vasculitis, and demyelination in sarcoid neuropathy
Source: Eur J Neurol. 2023 Oct 17;31(1):e16091. doi: 10.1111/ene.16091 (PMC11235865; doi:10.1111/ene.16091)
Supplement: Supplementary file 2 — Table S1. [file ENE-31-e16091-s002.pdf]

Supplementary Table1. *Conduction block and temoral dispersion*

Case	Conduction block	Temporal dispersion
1	Ulnar nerve	-
2	-	-
3	Ulnar nerve	Ulnar nerve
4	Peroneal nerve	-
5	Ulnar nerve (bilateral)	-
6	-	-
7	ND	ND
8	ND	ND
9	Peroneal nerve	-
10	-	-
11	-	-
12	Median and peroneal nerves	Tibial and peroneal nerves
13	Peroneal nerve	-
14	Ulnar and peroneal nerves	Ulnar nerve
15	-	-
16	Ulnar nerve	-
17	Ulnar nerve	-
18	Median, ulnar, and peroneal nerves	Median nerve

- = absent; ND = not determined.

The definition of conduction block or temporal dispersion was based on the motor nerve conduction criteria for chronic inflammatory demyelinating polyneuropathy proposed by the European Academy of Neurology and Peripheral Nerve Society [17].
